# Phonon laser in the coupled vector cavity optomechanics

**DOI:** 10.1038/s41598-017-17395-x

**Published:** 2018-01-10

**Authors:** Bao Wang, Hao Xiong, Xiao Jia, Ying Wu

**Affiliations:** School of Physics, Huazhong University of Science and Technology, Wuhan, 430074 P. R. China

## Abstract

We presented a method to control the intensity of a phonon-laser mode (the vibrational excitations of a mechanical mode) by adjusting the polarization of the pump light based on the experimentally achievable parameters, which provides an additional degree of freedom to control the phonon laser action. Due to orthogonally polarized modes of cavity, the polarization behavior of light field which describes it’s vector nature is introduced to control phonon laser action in our scheme. Compared with the traditional phonon laser scheme, polarization-related phonon laser in the coupled vector cavity optomechanics can be effectively controlled without changing other parameters of the device. This result provides an useful approach for acquiring polarization-related phonon laser by on-chip optical device.

## Introduction

It is well known that the generation of photon lasing requires coherent amplification of the stimulated emission of photons in a cavity with the gain medium. Similarly, several theoretical and experimental proposals were put forward to produce phonon laser via coherent amplification of stimulated emission of phonons in various physical systems, including ions^1^, the quantum dot^2,3^, semiconductors^4^, nanomechanics^5–7^, nanomagnets^8^, and others^9^. The research of phonon lasing has made remarkable progresses in a wide range of systems in recent years and phonon stimulated emission has been observed experimentally in cryogenic Al_2_O_3_:Cr^3+^ ^10–12^, Al_2_O_3_:V^4+^ ^13^, semiconductor superlattices^14^ and a single cooled Mg^+^ ion^15^.

Cavity optomechanics (COM)^16^, which devotes to describe the interaction between optical modes and mechanical oscillator via the radiation pressure^17,18^, provides a special platform for performing phonon laser action. Observation of sub-poissonian phonon lasing in a three-mode optomechanical system^19^ and the discovery of phonon laser in the ${\mathscr{P}}{\mathscr{T}}$ symmetric regime^20^ have received increased attention due to their important applications in high precision measurement. And a proposed approach exhibits time-dependent stimulated phonon field amplification when one of the cavities is added with optical gain medium^21^. Specifically, a significative regime of phonon lasing in a compound Whispering-Gallery-Microcavities (WGM) system that operates in close analogy to a two-level laser system^22^ has been reported. According to the regime, the transition frequency between the two nondegenerate optical supermodes is induced by a phonon field and the efficient optomechanical coupling leads to the generation of phonon laser. This method has also been used in other areas of research, such as researches on optomechanical quantum information processing with photons and phonons^23^ and enhanced quantum nonlinearities in a two-mode optomechanical system^24^. In addition, phonon lasing has been analyzed theoretically in a coupled optomechanical system^25^, and a phase-controlled ultralow-threshold phonon laser is proposed by using tunable optical amplifiers in coupled-cavity-optomechanical system^26^. Significantly, considering orthogonally polarized modes in a whispering-gallery-mode resonator have revealed important physical mechanism^27,28^. Recently, the concept of vector cavity optomechanics^29,30^ has been put forward where the polarization behaviour of light is introduced to achieve optomechanical control. Here, we study phonon laser in the coupled vector cavity optomechanics and tunable phonon laser action with remarkable features is observed. Since the system pumped by polarized light field operates in close analogy to a two-level laser system, the phonon laser is formed through the coherent amplification of the stimulated emission phonons arising from the interaction between photon and phonon. We show that the phonon laser action exhibits strong polarization dependence of the pump light in this system, which enables effective control of phonon laser, and the polarization-related phonon laser may make spectacular advances by on-chip optical architectures in the near future.

## Results

### Theoretical model and dynamical equations

**Figure 1:**
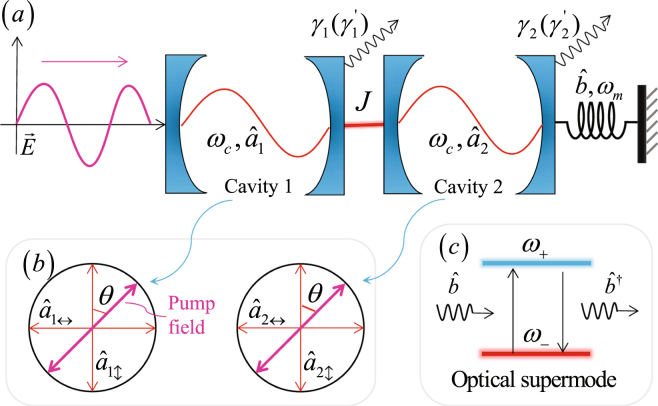
The vector cavity optomechancial system. (**a**) Schematic diagram of the coupled vector cavity optomechancial system, which is pumped by a linear polarized optical field. *J* is the coupling strength (also called photon-tunneling rate), which can be tuned by changing the distance between cavity 1 and cavity 2. (**b**) ${\hat{a}}_{1\updownarrow }\,({\hat{a}}_{1\leftrightarrow })$ and ${\hat{a}}_{2\updownarrow }\,({\hat{a}}_{2\leftrightarrow })$ are the TE (TM) modes of the cavity 1 and cavity 2. The included angle between the polarization of pump field and the vertical mode is *θ* for the two cavities. (**c**) Two-level phonon laser energy level diagram, the corresponding optical supermodes are coupled by phonons.

As shown in Fig. 1(a), the model we considered is composed of two coupled Fabry-Pérot cavities, and the two cavities are all high-Q optical microcavities. The right-hand cavity contains a movable mirror with effective mass *m* and eigenfrequency *ω*_*m*_, viz. an optical microcavity is directly coupled to an vector cavity optomechanical system. For the two Fabry-Pérot cavities, a group of orthogonal basis vectors of polarization ($\mathop{{e}_{\updownarrow }}\limits^{\rightharpoonup }$, $\mathop{{e}_{\leftrightarrow }}\limits^{\rightharpoonup }$) corresponding to transverse electric (TE) and transverse magnetic (TM) modes can be introduced^29^. The vector $\mathop{e}\limits^{\rightharpoonup }$ of any polarization states can be disintegrated as $\mathop{e}\limits^{\rightharpoonup }=\alpha {\mathop{e}\limits^{\rightharpoonup }}_{\updownarrow }+\beta {\mathop{e}\limits^{\rightharpoonup }}_{\leftrightarrow }$ with |*α*|^2^ + |*β*|^2^ = 1 [shown in Fig. 1(b)]. The two double-mode cavities can directly be coupled by the photon-tunneling rate *J*, which can be efficiently modulated by changing the spacing between the two cavities.

We stem from a generic optomechanical system, the coupled vector optomechanical system pumped by linearly polarized optical field can be described by the following Hamiltonian^31,32^:

1 $$\begin{array}{ccc}\hat{H} & = & {\hat{H}}_{0}-{\sum }_{j=\updownarrow ,\leftrightarrow }\hslash [J({\hat{a}}_{1j}^{\dagger }{\hat{a}}_{2j}+{\hat{a}}_{2j}^{\dagger }{\hat{a}}_{1j})+g({\hat{a}}_{2j}^{\dagger }{\hat{a}}_{2j})\hat{x}]\\  &  & +i\hslash [\sqrt{2{\gamma }_{1}}({\hat{a}}_{1\updownarrow }^{\dagger }{{\rm{\Omega }}}_{d\updownarrow }-{\rm{H}}.{\rm{c}}.)+\sqrt{2{\gamma }_{1^{\prime} }}({\hat{a}}_{1\leftrightarrow }^{\dagger }{{\rm{\Omega }}}_{d\leftrightarrow }-{\rm{H}}.{\rm{c}}.)],\\ {\hat{H}}_{0} & = & \frac{{\hat{p}}^{2}}{2m}+\frac{1}{2}m{\omega }_{m}^{2}{\hat{x}}^{2}+\hslash {\omega }_{c}{\sum }_{j=\updownarrow ,\leftrightarrow }({\hat{a}}_{1j}^{\dagger }{\hat{a}}_{1j}+{\hat{a}}_{2j}^{\dagger }{\hat{a}}_{2j}),\end{array}$$

where $\hat{p}$ and $\hat{x}$ are the momentum and position operators of the mechanical resonator, respectively. The coupling afforded by radiation pressure is described by the Hamiltonian $\hslash g{\sum }_{j}({\hat{a}}_{2j}^{\dagger }{\hat{a}}_{2j})\,\hat{x}\,(j=\updownarrow ,\leftrightarrow )$, where $\hslash$ is the reduced Planck constant, ${\hat{a}}_{1j}\,({\hat{a}}_{1j}^{\dagger })$ and ${\hat{a}}_{2j}\,({\hat{a}}_{2j}^{\dagger })$ are the annihilation (creation) operators of the orthogonal cavity modes of the cavity 1 and 2. The resonance frequency of the cavity mode is *ω*_*c*_ with the commutation relations $[{\hat{a}}_{1j},{\hat{a}}_{1j}^{\dagger }]=1$ and $[{\hat{a}}_{2j},{\hat{a}}_{2j}^{\dagger }]=1$. *g* = *ω*_*c*_/*L* is the single-photon optomechanical coupling rate, *L* is the cavity length, Ω_*d*_ is the amplitude of the pump field. For linearly polarized input field ${{\rm{\Omega }}}_{d}{e}^{-i\omega t}\mathop{e}\limits^{\rightharpoonup },\,{{\rm{\Omega }}}_{d}={e}^{i\theta }\sqrt{{P}_{in}/(\hslash \omega )}$ and *P*_*in*_ is the input power. $\mathop{e}\limits^{\rightharpoonup }$ is the unit vector of polarization of the input field, which can be expressed *a*s $\mathop{e}\limits^{\rightharpoonup }=a{\mathop{e}\limits^{\rightharpoonup }}_{\updownarrow }+b{\mathop{e}\limits^{\rightharpoonup }}_{\leftrightarrow }$, with *a* and *b* as the proj*e*ctions of $\mathop{e}\limits^{\rightharpoonup }$ at the vertical and horizontal modes, respectively. Using the included angl*e θ* between $\mathop{e}\limits^{\rightharpoonup }$ and the vertical mode as shown in Fig. 1(b), we obtain *a* = cos*θ* and *b* = sin*θ*. So, ${{\rm{\Omega }}}_{d\updownarrow }={{\rm{\Omega }}}_{d}\,\cos \,\theta {e}^{-i\omega t},\,{{\rm{\Omega }}}_{d\leftrightarrow }={{\rm{\Omega }}}_{d}\,\sin \,\theta {e}^{-i\omega t}$.

Based on the Hamiltonian, the Heisenberg-Langevin equations can be obtained to describe the evolution of the cavity field and the mechanical motion of the moving mirror. In our work, we neglect the quantum noise of the movable mirror and the cavity, and consider the mean values of all the operators. Considering the case of resonance, viz. *ω* = *ω*_*c*_, and involving loss of the system which is added phenomenologically, the Heisenberg-Langevin equations of motion in a rotating frame at *ω*_*c*_ can be written as follows:

2 $$\begin{array}{rcl}{\dot{\hat{a}}}_{1\updownarrow } & = & -{\gamma }_{1}{\hat{a}}_{1\updownarrow }+iJ{\hat{a}}_{2\updownarrow }+\sqrt{2{\gamma }_{1}}{{\rm{\Omega }}}_{d}\,\cos \,\theta ,\\ {\dot{\hat{a}}}_{2\updownarrow } & = & -{\gamma }_{2}{\hat{a}}_{2\updownarrow }+iJ{\hat{a}}_{1\updownarrow }+ig{\hat{a}}_{2\updownarrow }x,\\ {\dot{\hat{a}}}_{1\leftrightarrow } & = & -{\gamma }_{1}^{\prime} {\hat{a}}_{1\leftrightarrow }+iJ{\hat{a}}_{2\leftrightarrow }+\sqrt{2{\gamma }_{1}^{\prime} }\,{{\rm{\Omega }}}_{d}\,\sin \,\theta ,\\ {\dot{\hat{a}}}_{2\leftrightarrow } & = & -{\gamma }_{2}^{\prime} {\hat{a}}_{2\leftrightarrow }+iJ{\hat{a}}_{1\leftrightarrow }+ig{\hat{a}}_{2\leftrightarrow }x,\\ \ddot{\hat{x}}+{{\rm{\Gamma }}}_{m}\dot{\hat{x}}+{\omega }_{m}^{2}\hat{x} & = & \frac{\hslash g}{m}({\hat{a}}_{2\updownarrow }^{\dagger }{\hat{a}}_{2\updownarrow }+{\hat{a}}_{2\leftrightarrow }^{\dagger }{\hat{a}}_{2\leftrightarrow }),\end{array}$$

where *γ*_1_ and *γ*_2_ are the optical loss of the vertical mode in cavity 1 and cavity 2, similarly, ${\gamma }_{1}^{\prime}$ and ${\gamma }_{2}^{\prime}$ are the optical loss of the horizontal mode, respectively. Γ_*m*_ is the mechanical damping rate.

### The photons in the cavity 2

Using the semiclassical and mean-field approximations, e.g., $\langle \hat{{\rm{A}}}\hat{{\rm{B}}}\rangle =\langle \hat{{\rm{A}}}\rangle \langle \hat{{\rm{B}}}\rangle$, we can obtain the following steady-state solutions:

3 $$\begin{array}{rcl}{a}_{1\updownarrow ,s} & = & \frac{\sqrt{2{\gamma }_{1}}{{\rm{\Omega }}}_{d}\,\cos \,\theta ({\gamma }_{2}-ig{x}_{s})}{{\gamma }_{1}{\gamma }_{2}+{J}^{2}-ig{\gamma }_{1}{x}_{s}},\\ {a}_{2\updownarrow ,s} & = & \frac{iJ\sqrt{2{\gamma }_{1}}\,{{\rm{\Omega }}}_{d}\,\cos \,\theta }{{\gamma }_{1}{\gamma }_{2}+{J}^{2}-ig{\gamma }_{1}{x}_{s}},\\ {a}_{1\leftrightarrow ,s} & = & \frac{\sqrt{2{\gamma }_{1}^{\prime} }\,{{\rm{\Omega }}}_{d}\,\sin \,\theta ({\gamma }_{2}^{\prime} -ig{x}_{s})}{{\gamma }_{1}^{\prime} {\gamma }_{2}^{\prime} +{J}^{2}-ig{\gamma }_{1^{\prime} }{x}_{s}},\\ {a}_{2\leftrightarrow ,s} & = & \frac{iJ\sqrt{2{\gamma }_{1}^{\prime} }\,{{\rm{\Omega }}}_{d}\,\sin \,\theta }{{\gamma }_{1}^{\prime} {\gamma }_{2}^{\prime} +{J}^{2}-ig{\gamma }_{1}^{\prime} {x}_{s}},\\ {x}_{s} & = & \frac{\hslash g}{m{\omega }_{m}^{2}}({|{a}_{2\updownarrow ,s}|}^{2}+{|{a}_{2\leftrightarrow ,s}|}^{2})\mathrm{.}\end{array}$$

According to the expressions of ${a}_{2\updownarrow ,s}$, ${a}_{2\leftrightarrow ,s}$ and ${x}_{s}$, we can deduce the steady-state displacement of ${x}_{s}$ under the different input power *P*_*in*_, which denotes that the radiation and spring forces reach the balance.

**Figure 2:**
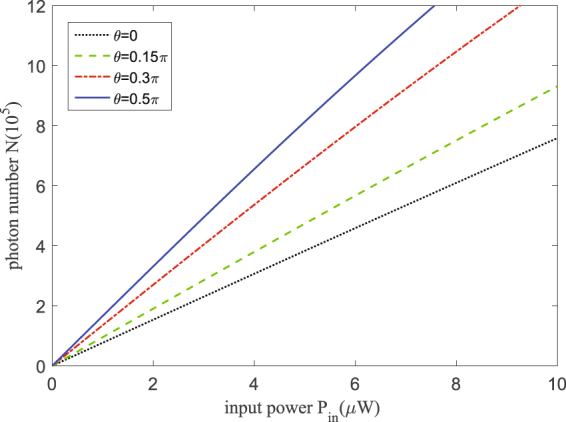
The photons number in cavity 2. The steady-state photons number *N* in cavity 2 vary with the input power. The parameters we take here are, *m* = 5×10^−11^ kg, *γ*_1_ = *γ*_2_ = 2π × 4.3 MHz, *γ*_1′_ = *γ*_2′_ = 2π × 3 MHz, Γ_*m*_ = 2.5 × 10^5^ Hz, *ω*_*m*_ = 23.4 MHz, *J* = *ω*_*m*_/2, respectively. The wavelength of the cavity field is 1550 nm. These parameters are derived from experiments^22^.

Figure 2 shows the steady-state values of the intracavity photons in the cavity 2, taking account of the feasibility of the experiment, we employ ${\gamma }_{1}\,=\,{\gamma }_{2}\,=\,2\pi \times 4.3\,{\rm{MHz}}$, ${\gamma }_{1}^{\prime} \,=\,{\gamma }_{2}^{\prime} \,=\,2\pi \times 3\,{\rm{MHz}}$. It is obvious that the photon number *N* increases linearly in the condition that pump power *P*_*in*_ ≤ 10 *μ*W, which can possess the same feature of passive COM systems^33^. Furthermore, the photons number can be modified by tuning the included angle *θ* between the polarization of pump field and the vertical mode. And as the *θ* rises, *N* is obtained giant enhancement, viz. the photon hopping effect is dramatically improved.

### Phonon laser in the vector cavity Optomechanical System

**Figure 3:**
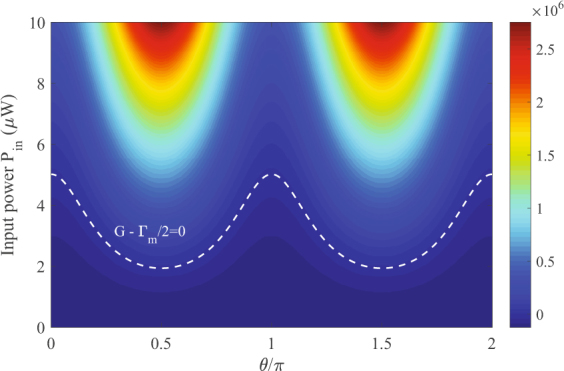
The gain of the mechanical mode. Calculation results of *G* −Γ_*m*_/2 varies with input power *P*_*in*_ and *θ*. We use the same parameters as in Fig. 1 and Δ*ω* = *ω*_*m*_.

In Fig. 3, we plot the gain of the mechanical mode $G^{\prime} =G-{{\rm{\Gamma }}}_{m}\mathrm{/2}$ as a function of the pump field *P*_*in*_ and the included angle *θ*. We observe a periodic behavior of the *G*′ from the figure, the period is *π*, and the maximum value of *G*′ is in the position of *θ* = 0.5*π*, which means when the pump light is horizontally polarized, viz. the effective optical decay rate is the minimum value, mechanical gain achieves the strongest value. The white dotted line describes $G^{\prime} =0$, and the figure indicates $G^{\prime}  > 0$ above this line. Significantly, the gain gets the maximum value at Δ*ω* = *ω*_*m*_.

**Figure 4:**
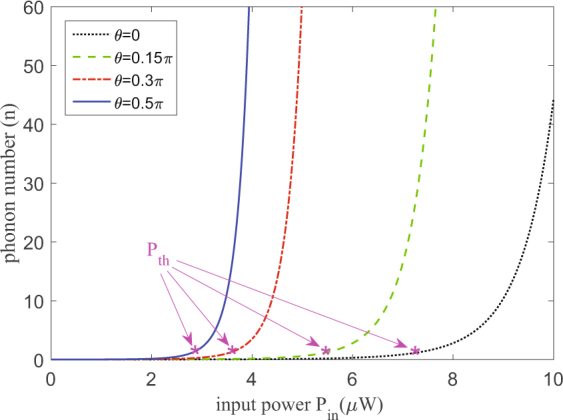
Phonon laser action in the vector regime. Calculation results of the stimulated emission of phonons *n* varies with input power *P*_*in*_ in the case of different angles. The phonon number *n* is expressed by *n* = exp[2(*G* − Γ_*m*_/2)/(Γ_*m*_/2)]. Setting the threshold condition *G* = Γ_*m*_/2, the asterisk denotes the threshold pump power *P*_*th*_ for the phonon laser under different angles. The threshold value of the *θ* = 0 is ~7 *μ*W, which can be lower for *θ* approaches *π*/2.

The Fig. 4 illustrates that the phonon number *n* is a function of the pump power *P*_*in*_ and for different angles. We can clearly infer that phonon gain is produced by coherent mechanical oscillation above the threshold for different polarization of pumping light, similar as ref.^22^. By taking advantage of the vector regime which contains orthogonally polarized modes in polarization nondegenerate cavities^28,30^, we find an additional degree of freedom to control the phonon laser action. Namely, tuning the polarization of pump light, the phonon number *n* can be modified without changing the other parameters, which can be easily realized in experiments.

## Discussion

In the main text, we elaborated that the polarization dependent phonon laser action is produced in the coupled vector cavity optomechanics which is pumped by linearly polarized light, where we introduce a group of orthogonal basis vectors of polarization in every Fabry-Pérot cavity. Moreover the input field with other polarization states, such as circular polarization and elliptical polarization, similar results may be obtained. In Fig. 2, we have studied the steady state of the vector regime, and the population of intracavity photons in cavity 2 is plotted under different input power. The optomechanical nonlinearity leads to mechanical gain *G*, and resulting in the emergence of phonon lasing action. As depicted in Fig. 4, when *θ* = 0, the threshold power is estimated as *P*_*th*_ ~ 7 *μ*W, agreeing well with the experiment^22^.

To conclude, we presented a method to control the intensity of the phonon laser by adjusting the polarization of the pump light. Our approach utilizes the polarization of light which is a fundamental property of optical fields, and we clearly show that the phonon laser action can be well controlled by tuning the polarization of the pump light. The strong polarization dependence of the phonon laser action may offer insight into the understanding of optomechanical interaction and find applications in manipulation of photon and phonon.

### Derivation of the phonon laser

In our system, the TE and TM modes of cavity 1 are pumped by input field together, and because the two modes are independent of each other, we can deal with the two paths respectively. Using the supermode operators ${\hat{a}}_{+j}=({\hat{a}}_{1j}+{\hat{a}}_{2j})/\sqrt{2}$, ${\hat{a}}_{-j}=({\hat{a}}_{1j}-{\hat{a}}_{2j})/\sqrt{2}$ ($j=\updownarrow ,\leftrightarrow$), we can deal with both paths in the same way. Looking at one of the two paths, the Fig. 1(c) describes the transition between the upper level *ω*_+_ and the lower level *ω*_−_ with *ω*_±_ = *ω*_*c*_ ± *J*, while simultaneously absorbing or emitting a phonon. The frequency *ω*_*m*_ of the mechan ical oscillator is equal to the transition frequency between the two supermodes, the eigenfrequencies *ω*_+_ and *ω*_−_ are split by 2*J*, and 2*J* = *ω*_*m*_. Similar to a two-level laser system, the stimulated emission of phonons will appear by achieving population inversion of the supermodes, the population inversion is correlated with the nonlinearity derived from the interaction induced by the radiation pressure of both TE and TM modes. Within the rotating-wave approximation (RWA), the interaction term of Hamiltonian can be converted to a simple form, i.e.,

4 $$-\hslash g({\hat{a}}_{2j}^{\dagger }{\hat{a}}_{2j})\hat{x}\to \frac{\hslash g{x}_{0}}{2}({\hat{p}}_{j}{\hat{b}}^{\dagger }+\hat{b}{\hat{p}}_{j}^{\dagger }),\,\,(j\,=\,\leftrightarrow ,\,\updownarrow )$$

where $\hat{x}={x}_{0}({\hat{b}}^{\dagger }+\hat{b})$ is the mechanical position operator, ${x}_{0}=\sqrt{\hslash \mathrm{/(2}m{\omega }_{m})}$ is the zero-point fluctuation amplitude of the mirror, and the operator $\hat{b}$ denotes the phonon mode. ${\hat{A}}_{j}={\hat{a}}_{-j}^{\dagger }{\hat{a}}_{+j}\,(j=\,\leftrightarrow ,\,\updownarrow )$ is the population inversion of the supermodes. And we make the another term $J({\hat{a}}_{1j}^{\dagger }{\hat{a}}_{2j}+{\hat{a}}_{2j}^{\dagger }{\hat{a}}_{1j})$ diagonalized, then we can rewrite the Hamiltonian $\hat{H}$ in Eq. (1) as^22^.

5 $$\begin{array}{rcl}\tilde{H} & = & \hslash {\omega }_{m}{\hat{b}}^{\dagger }\hat{b}+\sum _{j=\updownarrow ,\leftrightarrow }(\hslash {\omega }_{+}{\hat{a}}_{+j}^{\dagger }{\hat{a}}_{+j}+\hslash {\omega }_{-}{\hat{a}}_{-j}^{\dagger }{\hat{a}}_{-j})\\  &  & +\frac{\hslash g{x}_{0}}{2}\sum _{j=\updownarrow ,\leftrightarrow }(\hat{b}{\hat{a}}_{+j}^{\dagger }{\hat{a}}_{-j}+{\hat{a}}_{-j}^{\dagger }{\hat{a}}_{+j}{\hat{b}}^{\dagger })+{H}_{{\rm{\Omega }}},\\ {H}_{{\rm{\Omega }}} & = & i\hslash \sum _{\eta =+,-}[\sqrt{{\gamma }_{1}}({\hat{a}}_{\eta \updownarrow }^{\dagger }\,{\rm{\Omega }}{}_{d\updownarrow }-{\rm{H}}{\rm{.c}}{\rm{.}})+\sqrt{{\gamma }_{1}^{\prime} }({\hat{a}}_{\eta \leftrightarrow }^{\dagger }{{\rm{\Omega }}}_{d\leftrightarrow }-{\rm{H}}{\rm{.c}}{\rm{.}})]\end{array}$$

where *ω*_±_ = *ω*_*c*_ ± *J* are the optical frequencies of the supermodes and defined to be blue and red, respectively. The third term of the Hamiltonian $\hat{H}$ describes the destruction of one phonon leads to transition from the red supermode to the blue supermode and the reverse process. Using the Hamiltonian in Eq. (5), we can write the evolution equations for the mechanical mode and the operator ${\hat{A}}_{j}={\hat{a}}_{-j}^{\dagger }{\hat{a}}_{+j}$ as follows:

6 $$\begin{array}{l}\frac{{\rm{d}}{\rm{\Psi }}}{{\rm{d}}t}={\rm{\Lambda }}{\rm{\Psi }}+\xi ,\end{array}$$

where

7 $$\begin{array}{l}{\rm{\Lambda }}=(\begin{array}{ccc}-i{\omega }_{m}-{{\rm{\Gamma }}}_{m}\mathrm{/2} & -ig{x}_{0}\mathrm{/2} & -ig{x}_{0}\mathrm{/2}\\ ig{x}_{0}{\rm{\Delta }}{N}_{\updownarrow }\mathrm{/2} & -i{\rm{\Delta }}\omega -\gamma \mathrm{/2} & 0\\ ig{x}_{0}{\rm{\Delta }}{N}_{\leftrightarrow }/2 & 0 & -i{\rm{\Delta }}\omega -\gamma ^{\prime} \mathrm{/2}\end{array}),\end{array}$$

${\xi }={({{\rm{\Gamma }}}_{1}(t),{{\rm{\Gamma }}}_{2}(t),{{\rm{\Gamma }}}_{2}(t))}^{T},\,{\rm{\Delta }}{N}_{j}=({\hat{a}}_{+j}^{\dagger }{\hat{a}}_{+j}-{\hat{a}}_{-j}^{\dagger }{\hat{a}}_{-j}),$ ${\rm{\Psi }}={(\hat{b},{\hat{A}}_{\updownarrow },{\hat{A}}_{\leftrightarrow })}^{{\rm{T}}},{\rm{\Delta }}\omega ={\omega }_{+}-{\omega }_{-},\,\hat{A}={\hat{A}}_{\updownarrow }+{\hat{A}}_{\updownarrow },\,2\gamma \,=$ ${\gamma }_{1}+{\gamma }_{2},\,2\gamma ^{\prime} ={\gamma }_{1}^{\prime} +{\gamma }_{2}^{\prime}$ *, γ*_1, 2_ and *γ*_1, 2_ are the optical decay rates of the each cavity. Γ_1_(*t*) and Γ_2_(*t*) represent fluctuation operators corresponding to the supermodes and the mechanical resonator, we don’t care the impact of the Γ_1_(*t*), Γ_2_(*t*) here. And the driving field doesn’t work for the evolution of operator ${\hat{A}}_{j}$, so we neglect the term of *H*_Ω_ related to the supermodes ${\hat{a}}_{+j}$ and ${\hat{a}}_{-j}$. Δ*N*_*j*_ is the optical inversion operator. In order to solve Eq. (6) and find the mechanical gain, we assume that the decay rate *γ*, *γ*′ ≫ Γ_*m*_, and introduce the slowly varying amplitudes as follows:

8 $$\tilde{b}=\hat{b}{e}^{-i{\omega }_{m}t},\,{\tilde{A}}_{\updownarrow }={\hat{A}}_{\updownarrow }{e}^{-i{\rm{\Delta }}\omega t},\,{\tilde{A}}_{\leftrightarrow }={\hat{A}}_{\leftrightarrow }{e}^{-i{\rm{\Delta }}\omega t},$$

Substituting these into Eq. (6), we can get

9 $$\dot{\tilde{b}}=(-\frac{{{\rm{\Gamma }}}_{m}}{2}+G+i\,{\rm{\Omega }}{}_{m})\tilde{b}$$

where the mechanical gain *G* is given by

10 $$\begin{array}{rcl}G & = & {\rm{Re}}[\frac{{(g{x}_{0}\mathrm{/2)}}^{2}{\rm{\Delta }}{N}_{\updownarrow }}{i({\omega }_{m}-{\rm{\Delta }}\omega )+\gamma \mathrm{/2}}+\frac{{(g{x}_{0}\mathrm{/2)}}^{2}{\rm{\Delta }}{N}_{\updownarrow }}{i({\omega }_{m}-{\rm{\Delta }}\omega )+\gamma ^{\prime} \mathrm{/2}}]\\  & = & [\frac{{(g{x}_{0}\mathrm{/2)}}^{2}{\rm{\Delta }}{N}_{\updownarrow }\gamma \mathrm{/2}}{{({\omega }_{m}-{\rm{\Delta }}\omega )}^{2}+{(\gamma \mathrm{/2)}}^{2}}+\frac{{(g{x}_{0}\mathrm{/2)}}^{2}{\rm{\Delta }}{N}_{\updownarrow }\gamma ^{\prime} \mathrm{/2}}{{({\omega }_{m}-{\rm{\Delta }}\omega )}^{2}+{(\gamma ^{\prime} \mathrm{/2)}}^{2}}]\end{array}$$

which is related to the population inversion operator Δ*N*_*j*_ and the effective optical decay rates *γ* and *γ*′. In the process of calculation, we use the slow-varying approximation to solve the coupled equations, viz. comparing with the variation of ${\tilde{A}}_{j}\,,\,\tilde{b}$ can be seen as constant. The phonon number *n* is expressed by *n* = exp[2(*G* − Γ_*m*_/2)/(Γ_*m*_/2)] at *t* = [Γ_*m*_/2]^−1^.
